# Changing Media Coverage of Mental Illness and Suicide: Results from a Multi-Year Canadian Action Research Study

**DOI:** 10.1192/j.eurpsy.2023.91

**Published:** 2023-07-19

**Authors:** R. Whitley

**Affiliations:** Douglas Research Centre, McGill University, Montreal, Canada

## Abstract

**Abstract:**

Evidence suggests that the media plays an important role in shaping public beliefs and attitudes towards mental illness and people with mental illness. On the one hand, holistic and balanced portrayals that focus on treatments and recovery can help reduce stigma and prejudice by increasing knowledge and understanding. On the other hand, sensational and one-dimensional portrayals can create and perpetuate stigmas and stereotypes, which can contribute to prejudice, fear and social exclusion.

Related research indicates that the media can also influence suicidal behaviour. On the one hand, research indicates an increase in suicide mortality following romanticized, sensational and detailed media coverage of a suicide (the Werther effect). On the other hand, emerging research indicates a decrease in suicidal mortality following media coverage focused on suicide prevention, available resources and hopeful narratives (the Papageno effect).

This presentation will discuss an ongoing national action-research project that has been continuously funded since 2010, which aims to decrease inaccurate and stigmatizing coverage, while increasing hopeful and recovery-oriented coverage, in relation to mental illness and suicide. This will include discussion of (i) longitudinal results from a media monitoring project, examining coverage of mental illness from 2010 to the present; (ii) various educational initiatives targeted at journalists and journalism schools during the project; and (iii) an innovative citizen journalism programme aiming to produce alternative positive portrayals.

This presentation will be highly-relevant to people wanting to learn more about media coverage of mental health and suicide, and especially pertinent to people interested in conducting similar research elsewhere.

**Disclosure of Interest:**

None Declared

